# Effects of fluoridation of porcine hydroxyapatite on osteoblastic activity of human MG63 cells

**DOI:** 10.1088/1468-6996/16/3/035006

**Published:** 2015-06-02

**Authors:** Zhipeng Li, Baoxin Huang, Sui Mai, Xiayi Wu, Hanqing Zhang, Wei Qiao, Xin Luo, Zhuofan Chen

**Affiliations:** 1 Guanghua School of Stomatology, Hospital of Stomatology, Sun Yat-sen University, 56 LingYuan Road West, Guangzhou 510055, Guangdong, People’s Republic of China; 2 Guangdong Provincial Key Laboratory of Stomatology, 74 ZhongShan 2 Road, Guangzhou 510055, Guangdong, People’s Republic of China

**Keywords:** fluoridation, biological hydroxyapatite, porcine hydroxyapatite, osteoblasts, material-cell interactions

## Abstract

Biological hydroxyapatite, derived from animal bones, is the most widely used bone substitute in orthopedic and dental treatments. Fluorine is the trace element involved in bone remodeling and has been confirmed to promote osteogenesis when administered at the appropriate dose. To take advantage of this knowledge, fluorinated porcine hydroxyapatite (FPHA) incorporating increasing levels of fluoride was derived from cancellous porcine bone through straightforward chemical and thermal treatments. Physiochemical characteristics, including crystalline phases, functional groups and dissolution behavior, were investigated on this novel FPHA. Human osteoblast-like MG63 cells were cultured on the FPHA to examine cell attachment, cytoskeleton, proliferation and osteoblastic differentiation for *in vitro* cellular evaluation. Results suggest that fluoride ions released from the FPHA play a significant role in stimulating osteoblastic activity *in vitro*, and appropriate level of fluoridation (1.5 to 3.1 atomic percents of fluorine) for the FPHA could be selected with high potential for use as a bone substitute.

## 1. Introduction

Calcium phosphate-based ceramics have been widely used as bone substitutes in orthopedic and dental applications due to their similarity in composition with natural bone. From a chemical point of view, hydroxyapatite (HA) with the stoichiometric formula Ca_10_(PO_4_)_6_(OH)_2_ and a Ca/P ration of 1.67, is the material most similar to the inorganic part of bones and teeth [1]. During the past decades, much effort has been put into obtaining synthetic HA. Therefore, a variety of methods are available at present for this purpose, and synthetic HA has been widely studied, showing good biocompatibility and osteoconductivity [2, 3]. However, there are differences between synthetic HA and biological HA derived from animal bones. The latter has a chemical composition and structure closer to the inorganic part of human bone; it shows better metabolic activity, more dynamic response to environment [4, 5] and less intense inflammatory reactions than the synthetic one [6].

Fluorine is an essential trace element in bone tissue, which can promote the crystallization of calcium phosphate and further accelerate the mineralization during the process of bone formation [7, 8]. There is growing evidence that fluorine therapy for osteoporosis can directly stimulate bone formation and increase bone mass without prior bone resorption [8–10]. Moreover, fluorine is known to promote the proliferation and differentiation of bone-forming cells [11–14]. Thus, fluoride substitution for hydroxyapatite has been suggested as a method to further improve the osteogenic potential of the biomaterial. Fluorinated hydroxyapatite (FHA) has been reported to show comparable bioactivity and biocompatibility over HA [15]. Fluorine ions released from FHA increased collagen syntheses and alkaline phosphatase activity of osteoblasts [16]. And fluoride content in FHA has also been confirmed to have a significant impact on cell responses in terms of cell proliferation and differentiation [13, 17–19]. However, there is still no consensus regarding effects of fluoride incorporation in HA on cell responses. It was also reported that the cell proliferation rate and equivalent alkaline phosphatase (ALP) activity are lower with FHA compared to pure HA [20, 21]. Furthermore, a higher concentration of fluoride was suggested to reduce osteoconductivity and also to cause adverse effects, such as osteomalacia [13]. Therefore, it is necessary to control the level of fluoridation of HA to prevent these adverse effects and to achieve the best biological performance. Unfortunately, there is a lack of literature regarding the optimal ranges of fluoride substitution in terms of cell response [11, 13–18]. Furthermore, the previous studies [13–21] on FHA were based on the chemical synthetic version, and it is hard to find information about the physiochemical properties and biological performance of fluoridated biological HA.

Biological HA was obtained from porcine bone, and fluoridation was performed to fabricate fluorinated porcine hydroxyapatite (FPHA) by means of a straightforward chemical and thermal treatment used in our previous study [22]. The results confirmed the successful incorporation of fluoride into the lattice structure of this porcine hydroxyapatite (PHA) [22]. However, physiochemical properties are only part of the story for this novel biomaterial. Biological performance, such as cellular responses, osteoconductivity and osteoinductivity, is of fundamental concern. Therefore, the objective of the present study was to evaluate the biological performance of human osteoblastic-like MG63 cells on FPHA and further investigate the optimal degree of fluoridation for its potential use as a bone substitute. It was hypothesized that FPHA could release fluoride and a certain degree of fluoridation of FPHA could promote osteoblast responses.

## 2. Materials and methods

### 2.1. Preparation of FPHA

FPHA preparation consisted of two steps as described in our previous study [22]: PHA preparation and fluoride substitution. Briefly, PHA was prepared from cancellous porcine bone. First, macroscopic impurities were removed from the bone through boiling in an autoclave at 121 °C for 30 min. Then calcination was carried out at 800 °C for 2 h in air (heating rate: 10 °C min^−1^) in a muffle furnace (SGM6812BK, Sigma Furnace Industry, China). The thermally treated samples, known as PHA, were immersed in sodium fluoride aqueous solutions of varied fluoride concentrations (F: 0.25, 0.50, 0.75, 1.00 mol L^−1^) for 24 h. After chemical treatment in sodium fluoride solution, calcination was carried out at 700 °C for 3 h in air (heating rate: 10 °C min^−1^). Thermally treated samples, known as FPHA, were cooled down at room temperature, rinsed three times in deionized water to remove unbound sodium fluoride, and then dried at 80 °C for 12 h. Finally, bone blocks were ground into powder, and 200 mg of powder were compressed into a disk with a diameter of 8 mm and a thickness of 2 mm using a rotary tableting machine (ZP10A, TianQi Pharmaceutical Machinery Co., China). The FPHA disks were randomly classified into four groups: FPHA0.25, FPHA0.50, FPHA0.75, and FPHA1.00; these represented immersion in various fluoride concentrations: 0.25, 0.50, 0.75, and 1.00 mol L^−1^, respectively. The FPHAs served as the experimental groups, while PHA, immersed in deionized water, served as the control group.

### 2.2. Physiochemical properties

Changes in the crystallinity of the PHA and FPHA disks were examined using x-ray diffraction (XRD, D/MAX Ultima III, Rigaku, Japan). A diffracted beam graphite monochromator was used to produce Cu K*α* radiation at a scanning speed of 10° (2*θ*)/min. Diffraction patterns were compared to reference patterns of HA (JCPDS72-1243). The chemical composition of the PHA and FPHA disks was examined by energy dispersive spectroscopy (EDS, Quanta 400 FEG, the Netherlands). The functional groups of PHA and FPHA were identified using Fourier transform infrared spectroscopy (FTIR, Nicolet 6700, Thermo Fisher Scientific, USA). Infrared (IR) spectra were collected in transmittance mode with a scanning range of 400–4000 cm^−1^.

### 2.3. Fluoride ion release test

To mimic the cell culture conditions, first, all samples were pre-immersed in a cell culture medium for 24 h. Then, three samples for each group were immersed in 1.5 mL of cell culture medium and incubated at 37 °C for periods of up to 7 days. After predetermined periods of time, the total medium was replaced by a fresh medium, and 1 mL of the extracted medium was diluted with the same volume of ionic strength adjustment buffer solution for final evaluation. The fluoride ions concentration was determined by a fluoride-selective electrode (Ruosull, China) connected to an ion analyzer (Thermo Scientific, USA). Calibration was performed using a series of diluted standard fluoride solutions within a range of 0.05–20 ppm.

### 2.4. Cell culture with PHA and FPHA disks

Human osteoblast-like MG63 cells (human osteosarcoma cell line), obtained from cell banks of the Chinese Academy of Sciences (Shanghai, China), were used to evaluate the biological performance of the PHA and FPHA disks. MG63 cells were cultured under standard conditions (37 °C,with 5% CO_2_ atmosphere and 100% relative humidity) in Dulbecco’s modified Eagle’s medium (DMEM, Hyclone, USA) supplemented with 10% fetal bovine serum (Gibco, Australia) and 1% penicillin-streptomycin (Sigma-Aldrich, USA).

PHA and FPHA disks were fixed in a 48-well cell culture plate and irradiated by gamma rays (Co-60) at a dose of 25 kGy for 24 h for sterilization. All samples in the cell culture plate were pre-immersed in DMEM for 24 h before cell seeding. The cells, with a cell density of 2 × 10^4^/well were seeded onto the wells fixed with the disks and the cell culture medium was changed on alternate days. After pre-determined periods of time, a series of cell assays, including cell attachment, cytoskeleton, cell proliferation, alkaline phosphatase activity, and bone-related gene expression were conducted to determine cellular responses on the PHA and FPHA disks.

### 2.5. Cell attachment, morphology and cytoskeleton

Cell attachment and morphology were observed on day 1 and day 5 by scanning electron microscopy (SEM, FEI Quanta 400, the Netherlands) after cell seeding. The samples were rinsed three times with phosphate-buffered saline (PBS), and then fixed with 2.5% glutaraldehyde in PBS for 4 h. After dehydration in graded alcohols, samples were dried with CO_2_ by a critical point drier (HCP-2, HITACHI, Japan). Finally, the cells were gold-coated by sputtering, and cell attachment and morphology were observed by SEM.

The cytoskeleton of cells was evaluated on day 1 and day 5 by laser scanning confocal microscopy (LSCM, Zeiss Axio Z15, Carl Zeiss, Germany). At predetermined periods of time, the cells on FPHA were washed three times with PBS and fixed in 3.7% formaldehyde solution in PBS for 10 min at room temperature. Then 0.1% Triton-X100 PBS was added to increase cell membrane permeability, and 1% bovine serum albumin was added to reduce nonspecific background staining [23]. Finally, cells were stained with rhodamine-phalloidin 635 (Life technologies, Invitrogen, USA) for actin filaments and Hoechst 33342 (Sigma-Aldrich, USA) for nuclei.

### 2.6. Cell proliferation assay

The cells were allowed to attach to the PHA and FPHA disks for 1, 3, 5, and 7 days. The density of attached cells was assayed by following the standard method of cell counting kit-8 (CCK-8, Dojindo, Japan). Briefly, a 300 *μ*L cell culture medium and a 30 *μ*L CCK-8 solution were added to each well and the plates were placed in an incubator at 37 °C for 1 h. After incubation, 100 *μ*L solution from each well was transferred to a new 96-well cell culture plate, and colorimetric change was analyzed using a spectrophotometric microplate reader (GENios, Germany) at a wavelength of 450 nm, and the results were expressed as optimal density.

### 2.7. Alkaline phosphatase activity

Alkaline phosphatase activity was performed at 3, 7, 14, and 21 days after cell seeding onto the disks’ surface. At predetermined periods of time, the cells were detached from the disks with a 0.25% solution of trypsin and ethylenediaminetetraacetic acid. After centrifugation, the cell pellets were washed twice with PBS, and then lysed with 0.1% Triton X-100. Following three cycles of freezing/thawing, cells were centrifuged at 13 000 rpm for 5 min at 4 °C, and the supernatants were collected as cell lysates for ALP and total protein assay. The ALP activity assay was performed by testing the transformation of p-nitro-phenyl phosphate (p-NPP) into p-nitrophenol (p-NP). Briefly, 20 *μ*L supernatants were incubated with 80 *μ*L ALP reagent (Sigma-Aldrich, USA) and transferred into 96-well plates at 37 °C for 30 min. ALP activity was equivalent to the absorbance of p-NP, which could be measured at a wavelength of 410 nm using a spectrophotometric microplate reader (GENIOS, Germany). Then, a standard curve was made by a series of standard solutions of p-NP as references for measurement. Another part of the supernatants was used for total protein determination, which was performed using a bicinchoninic acid protein assay kit (Pierce, Thermo Scientific, USA). Finally, ALP activity was normalized to total protein content and expressed as *μ*mol p-NP/30 min mg^−1^ protein.

### 2.8. Bone-related gene expression by reverse transcription-polymerase chain reaction (RT-PCR)

The cells were allowed to attach to PHA and FPHA for 7 and 21 days and five disks served as one sample in order to isolate enough total RNA for evaluation. Briefly, at the predetermined time period, total RNA from cells was extracted using the Trizol reagent (Invitrogen, USA). The expressions of one housekeeping gene, *β*-actin (ACTB), and four osteogenic markers, Runt-related transcription factor 2 (Runx2), ALP, osteocalcin (BGLAP), and type I procollagen (Col-I), were analyzed by quantitative real-time RT-PCR using a sequence detection system (Biorad iQ5, USA). The primer pair sequences are shown in table 1. All samples were run in triplicates and the expression of target genes was normalized to ACTB; the *ΔΔ*Ct method was applied to analyze the PCR results [24].

**Table 1.** Primer sequences for polymerase chain reaction.

Gene	Accession	Primer sequences	Size (bp)
		Forward: 5′-CCCCAACTTCCTGTGCTC-3′	
Runx2	NM_001024630.3	Reverse: 5′-CTCAGCAGAATAATTTTCATCG-3′	149
		Forward: 5′-ACCATTCCCACGTCTTCAC-3′	
ALP	NM_000478.4	Reverse: 5′-TTGTAGCCAGGCCCATTG-3′	134
		Forward: 5′- CTTTGTGTCCAAGCAGGAG-3′	
BGLAP	NM_199173	Reverse: 5′-TCAGCCAACTCGTCACAGTC-3′	151
		Forward: 5′-AAGAGGCATGTCTGGTTCG-3′	
Col-I	NM_000088	Reverse: 5′-TAGGTGATGTTCTGGGAGGC-3′	145
		Forward: 5′-CCAACCGCGAGAAGATGA-3′	
ACTB	NM_001101	Reverse: 5′-CCAGAGGCGTACAGGGATAG-3′	97

### 2.9. Statistical analysis

For cell proliferation and osteogenic differentiation tests, data were presented as means ± standard deviations for n = 5. One-way analysis of variance, followed by post hoc multiple comparison tests using Bonferroni’s correction, was used to determine the differences of variables among groups and ‘*p* < 0.05’ was considered statistically significant difference. Statistical evaluations were performed with SPSS version 13.0.

## 3. Results

### 3.1. Physiochemical properties

**Figure 1.**
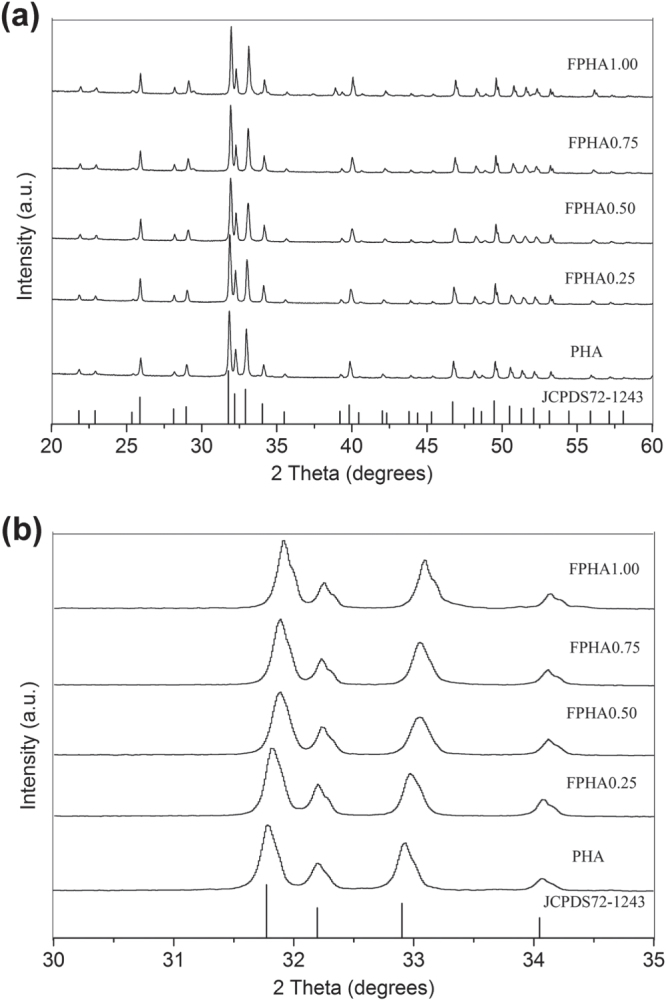
XRD patterns of the PHA and FPHA: (a) 20°–60°, similar patterns of PHA and FPHA; (b) 30°–35°, the enlargement displays shifts of apatite peaks before and after fluoridation.

The XRD signatures of PHA and FPHA (figure 1(a)) were in agreement with the stoichiometric reference HA pattern (JCPDS72-1243), indicating that PHA and FPHA were crystallized in the pure phase [22]. FPHA reflection peaks shifted toward higher diffraction angles with the increase in the level of fluoridation (figure 1(b)). The EDS results revealed that the main chemical component of PHA includes Ca, P, O, Na, and Mg. With the increasing level of fluoridation, increasing fluorine content from 1.50 to 6.67 atomic percents was detected (table 2).

**Table 2.** Chemical composition of PHA and FPHA samples by EDS.

	Atomic percentage (At. %)							
Sample	Ca	P	O	C	Na	Mg	F	Ca/P
PHA	18.81	12.62	58.31	9.15	0.64	0.47	0.00	1.49
FPHA0.25	17.68	11.95	59.53	8.24	0.58	0.52	1.50	1.48
FPHA0.50	18.17	12.03	59.14	6.77	0.81	0.61	2.47	1.51
FPHA0.75	17.94	11.64	57.96	8.12	0.77	0.45	3.12	1.54
FPHA1.00	18.38	12.09	53.34	7.36	1.85	0.31	6.67	1.52

**Figure 2.**
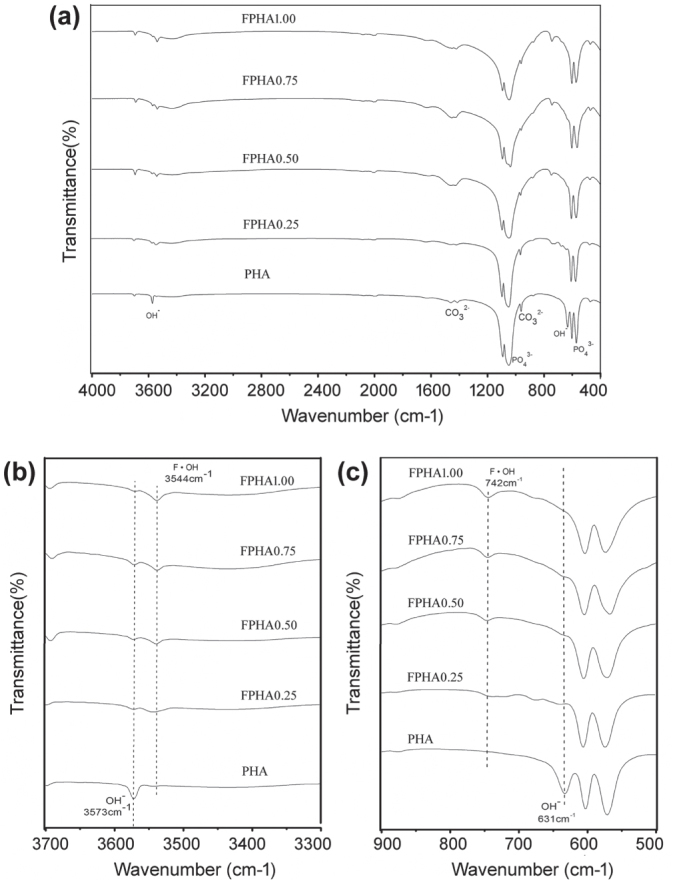
FTIR spectra of PHA and FPHA. Spectra are offset for clarity.

Absorption peaks corresponding to functional groups and the apatite phase were identified in the IR spectra of PHA and FPHA. In particular, functional groups 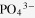 (1058 cm^−1^ and 569 cm^−1^), hydroxyls (OH, 631 cm^−1^ and 3573 cm^−1^), and 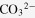 (1415 cm^−1^ and 1477 cm^−1^) [22] were identified by FTIR (figure 2(a)). Fluoride substituted for OH in the crystal structure of PHA after sodium fluoride immersion, and further thermal treatment was also confirmed: only one single band at 3573 cm^−1^ attributed to OH was identified for PHA. After fluoridation, the adsorption band attributed to OH stretching split into two bands at 3573 cm^−1^ and 3544 cm^−1^. Furthermore, with the increasing degree of fluoridation, the intensity of the OH characteristic band at 3573 cm^−1^ became weaker, while the other band at 3544 cm^−1^ attributed to hydrogen interacting with fluorine became stronger [25, 26]. Similarly, another major change in the FTIR spectra of FPHA was that the absorption band attributed to OH around 634 cm^−1^ disappeared, and was replaced by another band around 742 cm^−1^ attributed to OH interacting with fluorine [26, 27], which was further evidence for fluoride incorporation (figure 2(c)).

### 3.2. Fluoride ion release

**Figure 3.**
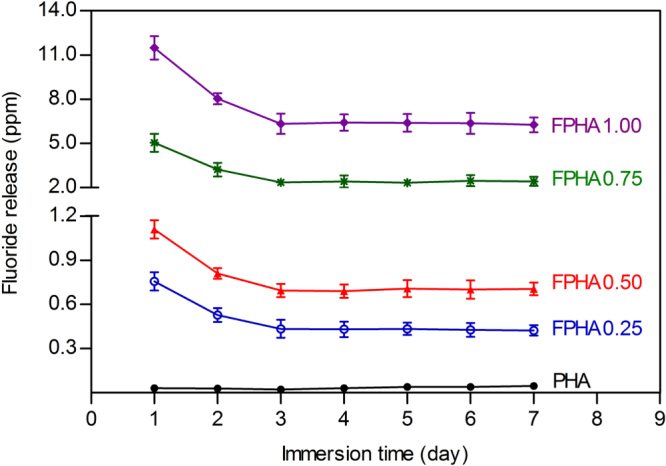
Concentration of fluoride ions released from PHA and FPHA to the cell culture medium.

Fluoride ion concentration in the cell culture medium was calculated from the standard calibration curve. Fluoride release was dose-dependent on the degree of fluoridation of FPHA, as compositions with a high fluoride content released more fluoride in the cell culture medium (figure 3). The released fluoride also decreased with continuous immersion time during the first 2 days, but then the concentration of fluoride remained constant from day 3 to day 7 for all FPHA groups.

### 3.3. Cell attachment and morphology

**Figure 4.**
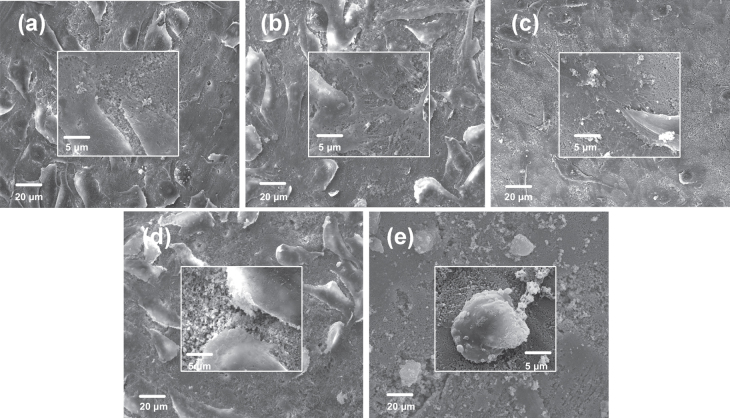
SEM images revealing the morphology of MG63 cells attached to the material surface after 1 day of culture: (a) PHA, (b) FPHA0.25, (c) FPHA0.50, (d) FPHA0.75, and (e) FPHA1.00. Insets show magnified views.

**Figure 5.**
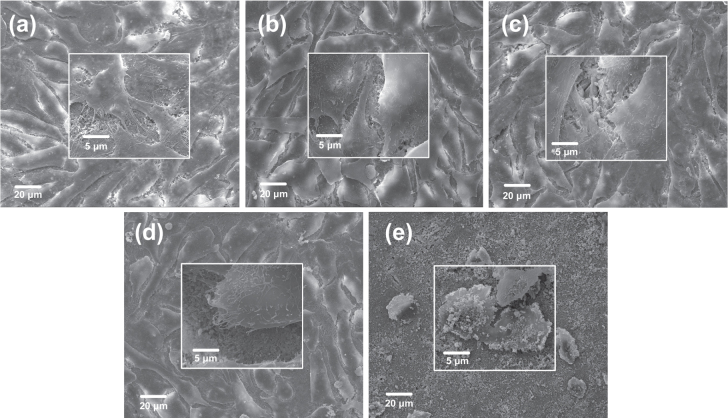
SEM images revealing the morphology of MG63 cells attached to the material surface after 5 days of culture: (a) PHA, (b) FPHA0.25, (c) FPHA0.50, (d) FPHA0.75, and (e) FPHA1.00. Insets show magnified views.

SEM was used to detect the morphology and assess the cytocompatibility of attached cells on PHA and FPHA surfaces after 1 and 5 days (figures 4 and 5). After 1 day of culture, cells were connected to each other and firmly attached to the surface for the PHA, FPHA0.25, and FPHA0.50 groups (figures 4(a)–(c)). In the high magnification view, cells exhibited typical osteoblast type, which appears cuboidal with many lamellipodia and filopodia extensions. However, fewer cytoplasmic extensions and filopodia on FPHA0.75 were evident when compared with the control (figure 4(d)). Moreover, compared with the other four groups, far fewer cells, mostly round-shaped without spreading, were observed on FPHA1.00 (figure 4(e)). After 5 days of culture, cells had undergone a significant spreading on the surface; colonized multilayered cells covered material surfaces, and numerous cells contacts were observed on PHA, FPHA0.25, FPHA0.50, and FPHA0.75 (figures 5(a)–(d)), indicating superior cell viability. However, there were still far fewer cells growing on FPHA1.00 (figure 5(e)). Furthermore, in a high magnification view, cells exhibited shrinkage, indicating that the FPHA1.00 surface was not cytocompatible.

### 3.4. Immunofluorescence and cytoskeletal observation

**Figure 6.**
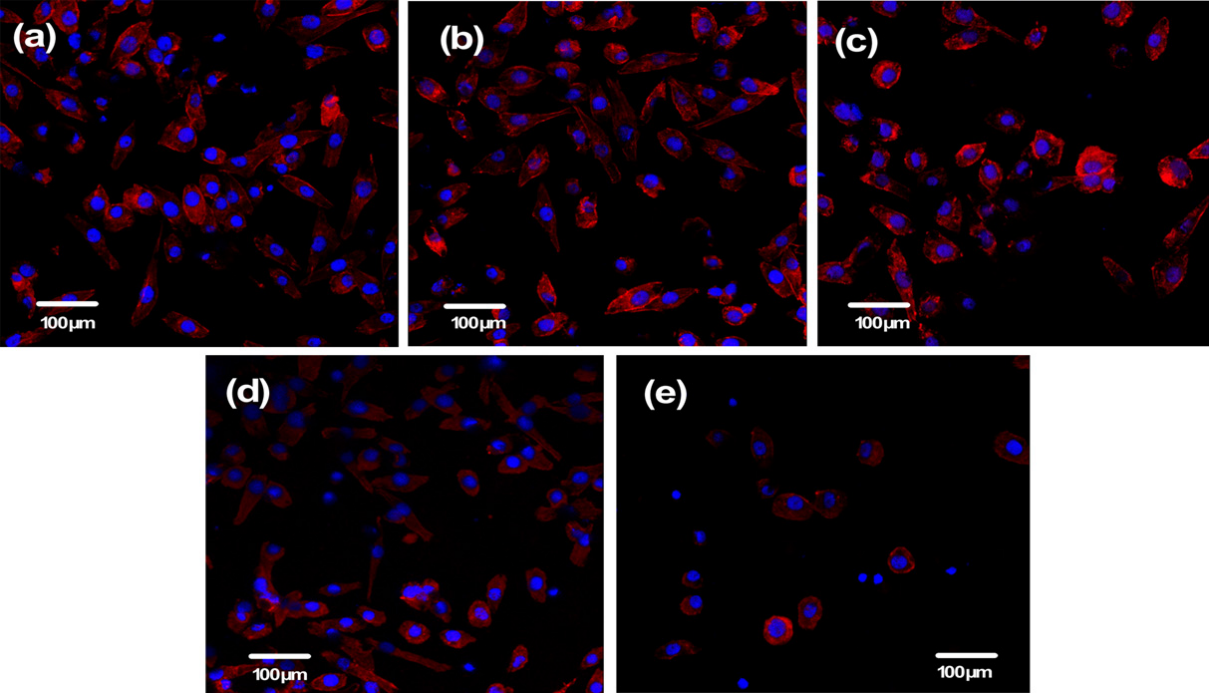
Confocal micrographs showing cytoskeleton of MG63 cells on the material surface after 1 day of culture: (a) PHA, (b) FPHA0.25, (c) FPHA0.50, (d) FPHA0.75, and (e) FPHA1.00. Cells were stained with rhodamine-phalloidin (red) for actin filaments and Hoechst 33342 (blue) for nuclei.

**Figure 7.**
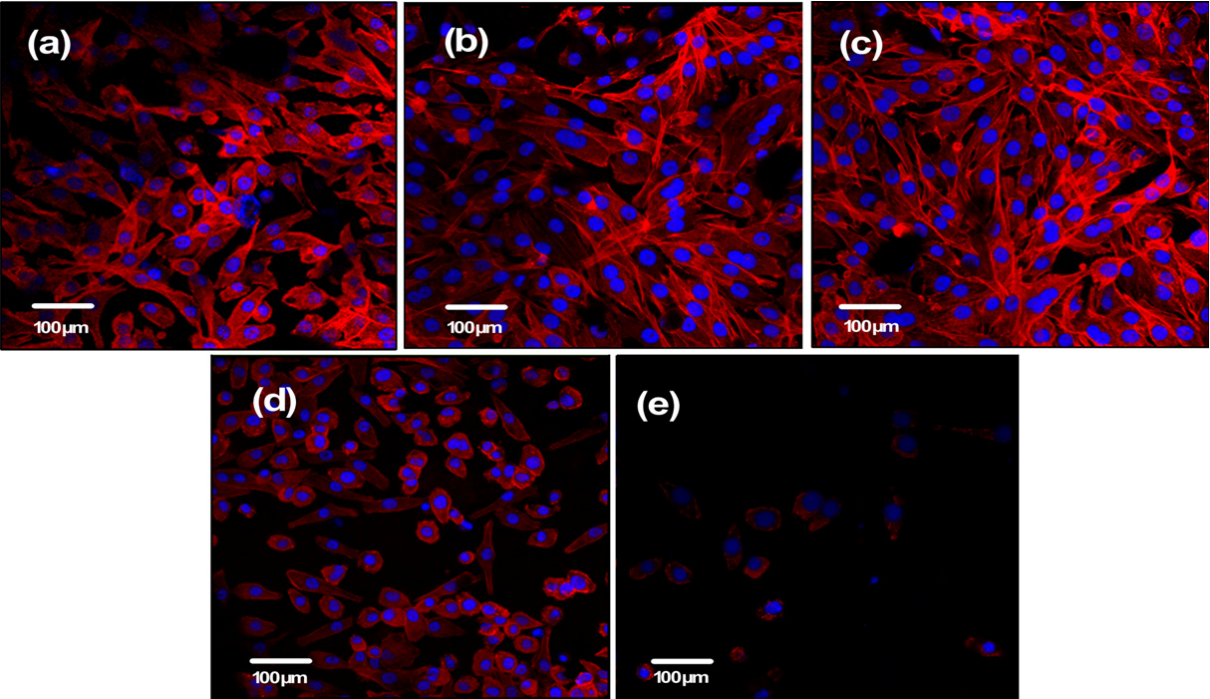
Confocal micrographs showing the cytoskeleton of MG63 cells on the material surface after 5 days of culture: (a) PHA, (b) FPHA0.25, (c) FPHA0.50, (d) FPHA0.75, and (e) FPHA1.00. Cells were stained with rhodamine-phalloidin (red) for actin filaments and Hoechst 33342 (blue) for nuclei.

Observation of the cytoskeleton, determined under LSCM by actin-staining with fluorescence-labeled phalloidin, was used to assess cell motility, spreading, and cell shape (figures 6 and 7). After 1 day of culture, cells were shown to firmly attach to the PHA and FPHA surfaces, and the cells on the PHA showed a network of formed stress fibers of normal filamentous morphology (figure 6(a)). The shape of the cells on FPHA0.25 and FPHA0.50 showed no difference when compared to the control, with clear longitudinal stress fibers (figures 6(b) and (c)). However, the actin cytoskeleton was less diffuse with less stress fiber formation in cells cultured on FPHA0.75 (figure 6(d)). Furthermore, there were far fewer attached cells on FPHA1.00 compared with the other four groups (figure 6(e)). After 5 days of culture, the cells proliferated rapidly and became confluent on PHA, FPHA0.25, FPHA0.50, and FPHA0.75 samples (figures 7(a)–(d)). Also, cells were shown to contact each other with cellular protrusions and extensions. However, cells attached on FPHA1.00 were still in the minority, with a shrunken cell shape (figure 7(e)).

### 3.5. Cell proliferation

**Figure 8.**
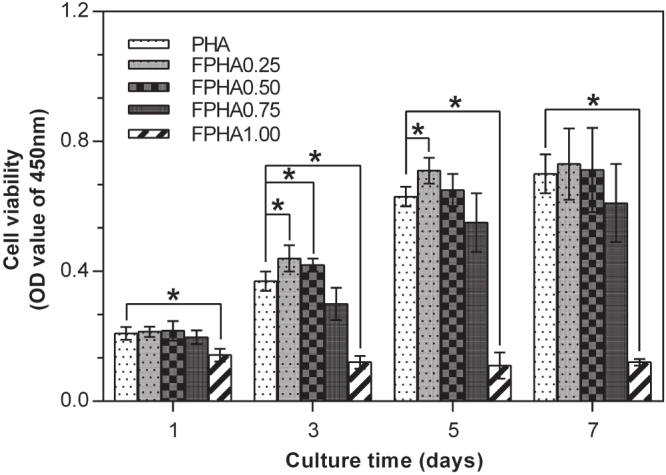
CCK-8 assay showing the proliferation of MG63 cells on days 1, 3, 5, and 7 cultured on PHA and FPHA surfaces. For clarity, only significant differences in comparison with the control are indicated on the graph (∗ indicated *p* < 0.05).

The MG63 cells proliferation assay by CCK-8 is shown in figure 8. At all the predetermined time periods, significantly far fewer cells were attached to FPHA1.00 than to the other groups. Furthermore, no growth in the number of cells was shown on FPHA1.00 from day 1 to day 7, indicating that the surface was not cytocompatible. Compared with the initial cell density, there was a significant increase in the amount of cells after 5 days of culture in the other four groups. Significantly more cells had grown on FPHA0.25 compared with the control PHA group by day 3 and day 5. Similarly, more cells had grown on FPHA0.50 compared with the control group by day 3 and day 5, while there was only a statistical difference between these two groups at day 3. By contrast, fewer cells attached to FPHA0.75 compared with the other three groups, but there was no statistical significant difference among them throughout the entire evaluation period. At day 7, the quantity of cells was almost equal to that of day 5 for each group, which may be because the whole cover of the material surface inhibited the cells’ continuous growth.

### 3.6. Alkaline phosphatase activity

**Figure 9.**
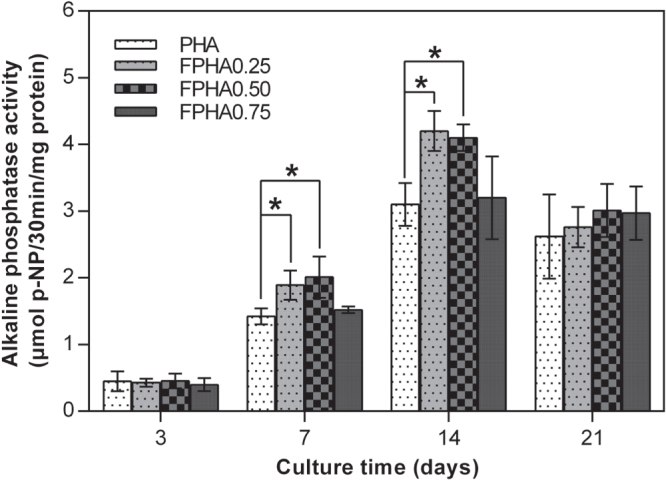
ALP activity of MG63 cells cultured on FPHA over the culture period. By day 7 and day 14, the ALP activity of the cell on FPHA0.25 and FPHA0.50 was significantly higher than that on the control group (∗ indicated *p* < 0.05).

ALP activity is a reliable indication of the early osteoblastic activity of cells. Since the quantity of cells grown was so small, the ALP activity of cells on FPHA1.00 was not evaluated. The amount of ALP production increased with the incubation time from day 3 to day 14, and then decreased from day 14 to day 21 for MG63 cells cultured on PHA and FPHA (figure 9). Significantly higher ALP activity of MG63 cells was shown on FPHA0.25 and FPHA0.50 compared with that on the control at days 7, 14, and 21. However, there is statistical difference only on day 7 and day 14.

### 3.7. Bone-related gene expression

**Figure 10.**
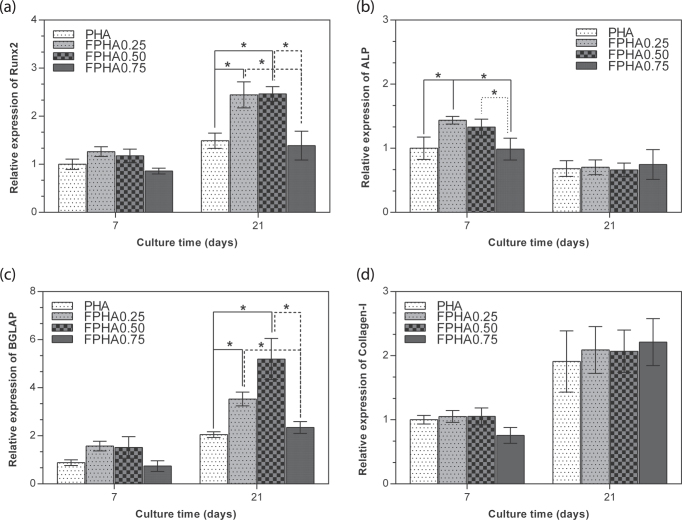
Comparison of expression levels of osteogenic marker genes by real-time RT-PCR. MG63 cells were cultured on PHA and five disks for 7 and 21 days. Messenger RNA levels of (a) Runx2, (b) ALP, (c) BGLAP, and (d) Col-I at different stages were measured. The ACTB gene was used as an internal control. Error bars indicate standard error of the mean (∗ indicated *p* < 0.05).

The bone-related gene expression levels of the cells cultured on PHA and FPHA for 7 and 21 days were analyzed using real-time RT-PCR, as shown in figure 10. For cells on all the samples, there was the same trend evident for bone-related gene expression, with a higher expression of Runx2, BGLAP, and Col-I on day 21 than on day 7; and a lower expression of ALP on day 21 than on day 7. Runx-2 was significantly up-regulated on FPHA0.25 and FPHA0.50 at day 21 with respect to the FPHA0.75 and control PHA groups (figure 10(a)). Meanwhile, ALP was significantly up-regulated on FPHA0.25 and FPHA0.50 at day 7, with respect to the control (figure 10(b)). Transcription levels of BGLAP were also significantly up-regulated on day 21 on FPHA0.25 and FPHA0.50 at day 21, with respect to the control (figure 10(c)). However, the expression levels of Col-I were quite similar among the four groups at day 7 and day 21 (figure 10(d)).

## 4. Discussion

The dissolution behavior of fluoridated HA is one of key physiochemical properties affecting *in vitro* cellular responses. Fluoride ions were slowly released from FPHA in a controlled manner depending on the level of fluoridation since most of the fluoride was incorporated into the lattice structure, which was confirmed in the x-ray photoelectron spectroscopy (XPS) results of our previous study [22]. The results of FTIR in the present study further confirmed that fluoride substituted for hydroxyl groups in the apatite crystal structure. Also, the fluoride substituted for hydroxyls causes the shift of the reflection in the XRD pattern [25, 27]. Previous studies on the dissolution behavior of synthetic FHA were mainly based on deionized water [16, 18, 26], physiological saline solution [17, 25] and simulated body fluid [28], which could not simulate the *in vivo* environment of implant biomaterial appropriately. A cell culture medium, which contains various ions, amino acids, and proteins more similar to body fluid, has been suggested to be a better source to mimic the dissolution behavior of biomaterial in a physiological environment [7, 14].

FPHA with a higher level of fluoridation released a larger amount of fluoride during the cell culture medium immersion tests in the present study. As we have discussed previously, there is little literature reporting the dissolution behavior of fluoridated biological HA. Compared with several previous studies on synthetic FHA [18, 25], FPHA in the present study is shown to have improved ability to release fluoride. This could be explained on the basis of carbonate groups in the lattice structure of biological HA [22], which may further lead to an increase of solubility compared with synthetic HA [10, 25].

The amount of released fluoride ions from FPHA decreased with continuous immersion time during the first 2 days, and then became constant from day 3 to day 7. This fluoride release behavior could be explained as follows: there are two classifications of fluoride of FHA—namely, ‘firmly bound fluoride,’ which is associated with fluoride incorporated into apatite lattice, and ‘loosely bound fluoride,’ which is generated and bound on the surface [19, 28]. During the first 2 days’ immersion, the majority of the ‘loosely bound fluoride’ and part of the ‘firmly bound fluoride’ were first released into the medium. Then, from day 3 to day 7, mainly the ‘firmly bound fluoride’ from the FPHA was released into the medium until dissolution equilibrium was achieved, which led to the constant release of fluoride ions. However, the amount of fluoride released was still not directly proportional to the degree of fluoridation, especially with regard to FPHA1.00, which showed a superior ability to release fluorine ions compared with the other groups. For FPHA1.00, the concentration of 1 mol L^−1^ sodium fluoride used in the chemical treatment during the fabrication process was at the maximum of its solubility, which makes sodium fluoride precipitates easier to bond to the PHA surface. The porous structure of PHA bone blocks makes it difficult to remove these sodium fluoride precipitates using a deionized water rinse, which could be the reason why FPHA1.00 contained a larger amount of sodium and also released a much larger amount of fluoride than the other FPHA samples, finally leading to cytotoxicity. Considering this, for chemical treatment of HA in a sodium fluoride solution, a relatively lower concentration than the saturated concentration would be more appropriate.

Fluorine is an essential trace element for bone formation. It is widely accepted that each trace element has an acceptable range of concentration, in which the element functions can maintain normal metabolic reactions [25]. When a trace element concentration of body fluid is close to the upper limit of the acceptable range, it is suggested that the element-dependent biological reaction can be improved compared with the normal state without causing any detrimental effects [25]. In the present study, the fluoride concentration released by FPHA0.25 and FPHA0.50 in the cell culture medium kept stable at 0.44 and 0.71 ppm (440 *μ*g L^−1^ and 700 *μ*g L^−1^), which is 10.1–16.2 folds of the upper range (43.7 *μ*g L^−1^) of body fluid of normal adults [25, 29] and also lower than the estimated toxic level of the serum fluoride concentration (950 *μ*g L^−1^) [30]. However, FPHA0.75 and FPHA1.00 constantly released fluoride at 2.30 and 6.42 ppm from day 3 to day 7, which is about 2.4 and 6.8 times higher than the estimated toxic level of the serum fluoride concentration [30]. These results indicated that an appropriate degree of fluoridation of FPHA, corresponding to FPHA0.25 and FPHA0.50 with a fluorine atomic percentage of 1.50 to 2.47, may be suitable for further investigation.

It is generally agreed that fluoridation of hydroxyapatite leads to a decrease of solubility, and it was reported that FHA releases less calcium than pure HA [18, 31]. Calcium ions have been shown to activate calcium channels and stimulate cell response [32], and the enhancement of osteoblastic potential has also been confirmed due to the calcium ions released from hydroxyapatite [33]. Considering this, it is logical to expect that PHA with a higher solubility than FPHA should stimulate cell response. However, the cells’ proliferation and osteogenic differentiation on FPHA0.25 and FPHA0.50 were improved compared with that on PHA in the present study, indicating that the enhancement should be due to the amount of fluoride released from FPHA during the cell culture process.

The SEM, LSCM, and cell proliferation results were in agreement with each other, which indicated that fluoridation affected cellular responses. For FPHA0.25 and FPHA0.50, the attachment, morphology, and cytoskeleton of cells were not significantly influenced by the fluoridation, as shown by SEM and LSCM studies, compared with PHA. However, the beneficial effect of fluoridation was manifested in the cell viability, ALP activity, and bone-related gene expression. An improved cell proliferation rate was shown on FPHA0.25 and FPHA0.50 compared with PHA at day 3 and 5. Reduced cell viability was shown on FPHA1.00 during the whole cell culture period, suggesting that a lower fluoride content might stimulate cell proliferation and a higher fluoride content might induce cytotoxicity, which is in agreement with several previous studies [13, 17, 19]. Since the quantity of cells grown was so small, the ALP activity and bone-related gene expression were not evaluated on FPHA1.00. ALP activity is a sensitive and early osteogenic differentiation marker of osteoblasts. The ALP activity increased with the culture time from day 3 to day 14, and then decreased from day 14 to day 21 for MG63 cells cultured on PHA and FPHA, indicating that MG63 cells stepped into the next differentiated stage from day 14 to day 21 [17]. The increase of ALP activity was shown on day 7 and day 14 on FPHA0.25 and FPHA0.50 compared with that on PHA, suggesting a fast differentiation of MG63 cells on the surface of fluoridated materials. Regarding transcription levels of osteogenic markers, ALP was significantly up-regulated on day 7, while Runx2 and BGLAP were also both significantly up-regulated on day 21 for FPHA0.25 and FPHA0.50, with respect to the control PHA group. ALP is an important early differentiation marker; Runx2 is a key transcription factor for osteoblast differentiation; BGLAP is the encoding gene for osteocalcin, which is a potent promoter protein for the nucleation of biominerals [34]. Therefore, these results clearly show that both FPHA0.25 and FPHA0.50 have the ability to stimulate the osteoblastic differentiation of cells. Based on these results, we conclude that an appropriate level of fluoridation of FPHA stimulates the osteoblastic proliferation and osteoblastic differentiation at the mRNA level as well as the ALP protein synthesis.

In summary, compared with PHA, FPHA0.25 and FPHA0.50 presented significant enhancement with regard to cell proliferation rate, ALP activity, and bone-related gene expressions, suggesting that fluoride released from FPHA plays an active role in stimulating cell proliferation and osteoblastic differentiation. There were no significant differences between FPHA0.75 and PHA in terms of cell viability, ALP activity, and bone-related gene expressions. However, for the higher fluoridation FPHA1.00, far fewer cells could be detected, indicating that higher fluoride may be cytotoxic. We, therefore, conjecture that an appropriate level of fluoridation (1.50 to 3.12 atomic percents of fluorine) of biological hydroxyapatite may be a suitable option for FPHA fabrication. However, more research, especially *in vivo* studies, should be conducted to evaluate the biological and biomechanical properties of these FPHA ceramics.

## 5. Conclusions

Samples of FPHA with different concentrations of fluoride were prepared by straightforward chemical and thermal treatment. Fluoride was confirmed to incorporate into the apatite lattice structure of FPHA by XRD and FTIR. FPHA with a fluoride content of 1.50 to 3.12 atomic percents can slowly release a certain amount of fluoride in a cell culture medium, and promote MG63 cell proliferation and osteogenic differentiation.
